# Long-Term Complications of Tracheoesophageal Voice Prosthesis

**DOI:** 10.3390/jcm13071912

**Published:** 2024-03-26

**Authors:** Maria Rita Bianco, Vincenzo Saita, Federico Occhiuzzi, Domenico Michele Modica, Daniele Latella, Alfio Azzolina, Mario Galfano, Eugenia Allegra

**Affiliations:** 1 Department of Health Science, University of Catanzaro, 88100 Catanzaro, Italy; mrbianco@unicz.it (M.R.B.); federico.occhiuzzi@studenti.unicz.it (F.O.);; 2 Otolaryngology Unit, Cannizzaro Hospital, 95100 Catania, Italy; vincenzosaita@yahoo.it (V.S.); alfioazzolina@gmail.com (A.A.); 3 Otolaryngology Unit, Villa Sofia-Cervello Hospital, 90146 Palermo, Italy; domenicomichelemodica@gmail.com (D.M.M.); mariogalfano@tiscali.it (M.G.)

**Keywords:** total laryngectomy, tracheoesophageal prosthesis, voice rehabilitation, laryngeal cancer, tracheoesophageal prosthesis complications

## Abstract

**Background**: The aim of our multicenter retrospective study was to evaluate the long-term complications associated with primary and secondary tracheoesophageal puncture (TEP) in patients who underwent total laryngectomy (TL) for laryngeal cancer and were subsequently rehabilitated to phonatory function with tracheoesophageal speech (TES). **Materials and Methods**: To evaluate the long-term outcomes and complications of TEP, the following data were collected: mean time of prosthesis replacement, mean time of onset of complications, type of complications, and type of failure. **Results**: Complications occurred in 18 out of 46 patients (39.2%) with primary TEP and in 10 out of 30 patients (33.4%) with secondary TEP, out of a total of 76 enrolled patients. Common complications included prosthesis leakage, fistula leakage, granulation, and prosthesis extrusion. Prosthesis replacement due to fistula leakage or prosthesis extrusion was observed exclusively in the group of patients with primary TEP. Among the 28 patients (35.7%) who experienced complications, rehabilitation with TEP failed in 10 cases, primarily due to abandonment and spontaneous fistula closure. **Conclusions**: TEP, both primary and secondary, represents a valid option for vocal rehabilitation in patients undergoing TL. However, identifying prognostic factors that could influence the success of TEP would be beneficial to allow a targeted rehabilitation process.

## 1. Introduction

Laryngeal cancer represents 30–40% of head and neck malignancies [1]. According to the latest available data, in 2020, there were approximately 185,000 new cases of laryngeal carcinoma worldwide, and the disease was responsible for 99,840 deaths [2]. The type of treatment depends on the stage of the disease at diagnosis.

Transoral laser microsurgery, open partial horizontal laryngectomy, and radiotherapy are treatment modalities aimed at preserving organ function while maintaining oncological radicality [3,4,5]. However, to date, most patients affected by laryngeal carcinoma are diagnosed with an advanced stage of the disease, requiring total laryngectomy (TL). TL surgery has significant psychophysical and social consequences for the patient’s quality of life [6]; this is attributed to the immediate loss of phonation, resulting from the removal of the larynx, and to alterations in respiratory and olfactory functions due to the permanent separation of the upper from the lower airways, which consequently results in a disconnection between the airways and the mouth and nose. Voice deprivation is perhaps the most relevant limiting factor in social relationships, increasing feelings of solitude and tending to drive individuals into social isolation. Recovery of phonatory function can be achieved using esophageal speech (ES) or with tracheo-oesophageal speech (TES). In both methods of voice rehabilitation, an internal substitute sound source is placed in the pharyngoesophageal segment. Tracheoesophageal puncture (TEP) was introduced in the 1980s for voice rehabilitation following TL and has since become the gold standard for voice restoration following TL, and voice rehabilitation with TES has been the preferred method in recent years because it allows faster vocal rehabilitation and a better voice [7], even if it involves a constant commitment for the patient in managing the prosthesis. TEP can be performed as a one-time procedure with TL (primary TEP) or as a secondary procedure at a later stage (secondary TEP). It appears to be clear that primary TEP avoids the need for a second surgery and provides faster speech rehabilitation after TL. However, no consensus has been reached on whether one approach is better than the other, and, moreover, most of the literature has focused on voice-related issues [8]. The purpose of our multicenter retrospective study was to perform an assessment of complications associated with primary and secondary TEP.

## 2. Materials and Methods

This retrospective multicenter study was conducted on previously untreated patients who underwent TL and TEP. The study participants were recruited during follow-up visits from 1 December 2022 to 31 January 2023 at the Otolaryngology Unit, Department of Health Science, University of Catanzaro; Otolaryngology Unit, Cannizzaro Hospital, Catania; Otolaryngology Unit, University of Catania; and Otolaryngology Unit, A.O. Riuniti Villa Sofia—Cervello of Palermo, Italy. Patients with locoregional recurrence or distant metastasis, and patients who refused to participate in the study were excluded from the research. We also excluded patients with surgical complications. All patients were informed about the study’s purpose before providing written informed consent. For each patient, clinical and anamnestic data, including age at the time of surgery, sex, follow-up period, tumor classification, node classification, neck dissection, chemo and/or radiotherapy, comorbidities, alcohol consumption, and type of TEP, were collected in a database. Patients who reported consuming more than 50 cl/day regularly were categorized as alcohol drinkers. The stage was determined in accordance with the 8th edition of the Tumor–Node–Metastasis (TNM) classification established by the American Joint Committee for Cancer [9]. Primary TEP was performed simultaneously with laryngectomy, with a puncture made using a trocar approximately 1.5–2 cm below the tracheal edge, while utilizing a pharyngeal protector to safeguard the posterior pharyngeal wall. A guide wire was then inserted for retrograde release of the prosthesis. Secondary TEP was conducted at a later stage after laryngectomy. A puncture was performed using an endoscopic guided retrograde technique under general anesthesia, employing a rigid esophagoscope and Provox insertion kit. All patients were fitted with Provox (ATOS Medical) prostheses. Prosthesis replacement was carried out with an anterograde approach, using a disposable insertion tool included in the new prosthesis kit. Furthermore, all patients were included in an oncological follow-up protocol, which involved periodic checks from ENT specialists and speech-therapy specialists.

### 2.1. Long-Term Tracheoesophageal Puncture Outcomes

To evaluate the long-term outcomes and complications of TEP, the following data were collected: mean time of prosthesis replacement, mean time of onset of complications, type of complications, and type of failure.

### 2.2. Statistical Analysis

A statistical analysis was performed using the MedCalc software version 9.0 (v 9.0; MedCalc Software bvba, Ghent, Belgium). The data collected included means, medians, and standard deviations. Pearson’s chi-square and/or Fisher’s exact test were used to identify differences in demographic and clinicopathologic data between cohorts. The paired sample t test was utilized to determine the mean difference between paired observations. A Kolmogorov–Smirnov test was used to assess the normal distribution of continuous variables. The Mann–Whitney U test for independent samples was applied to analyze group differences. All tests were two-tailed, and significance was set at *p* < 0.05.

## 3. Results

A total of 76 patients were included in the study, with 10 being female and 66 male. The mean age at diagnosis was 64.52 ± 11.3 standard deviation (SD) years, and the mean follow-up time was 42.42 ± 18 SD months. According to the clinical TNM (cTNM) classification, 40 lesions (52.6%) were staged as T3, and 36 (47.4%) as T4. The lymph node clinical status was classified as N0 in 39 patients and N+ in 37. A total of 69 out of 76 patients (90.7%) underwent neck dissection. Additionally, 32 of the 76 patients underwent surgery plus radiotherapy, while 15 underwent chemotherapy (Table 1). Of the total, 57 out of 76 patients (75%) had comorbidities. Moreover, 55 out of 76 patients (72.3%) were non-drinkers or consumed less than 50 cl/day, and 21 consumed more than 50 cl/day. Furthermore, 46 out of 76 patients (60.5%) had primary TEP, whereas 30 (39.5%) had secondary TEP. Statistically significant differences were found only in terms of follow-up time and age when comparing the clinical anamnestic data between the two groups, primary TEP vs. secondary TEP. (Table 2). All patients have undergone implantation of the Provox (Atos Medical) voice prosthesis. During the follow-up period, 28 out of 76 patients (36.8%) experienced TEP complications after a mean time of 3.9 ± 6.9 SD months. Complications occurred in 18 out of 46 patients (39.2%) with primary TEP and in 10 out of 30 (33.4%) with secondary TEP. In 51 patients, the voice prosthesis was replaced after a mean time ≤ 3 months, while in 25 it was replaced after a mean time of >3 months. There was no significant difference regarding the time of prosthesis replacement between primary and secondary implants (Table 3). Table 4 reports the type of complication that occurred in the 28 patients. The most frequent types of complications in a total of patients were prosthesis leakage (32.2%), and granulations (17.8%). No statistically significant difference was found regarding the occurrence of complications between patients with primary TEP and secondary TEP. Prosthesis replacement due to fistula leakage or prosthesis extrusion occurred only in the group of patients with primary TEP.

In 35.7% of cases (10 out of 28 patients), the complication did not resolve, leading to TEP rehabilitation failure. Among these cases, in four (40%) instances the fistula closed spontaneously, while in another four (40%) cases, patients abandoned rehabilitation due to aphonia or because they did not accept their voice. Additionally, surgical closure was necessary in two cases (20%) due to recurrent perifistular granulations and frequent prosthesis extrusion, respectively. Overall, failure was detected in 10 out of the total 76 patients (13.1%), with no significant differences observed between primary TEP (6/46, 13.0%) and secondary TEP (4/30, 13.3%). We stratified the clinical anamnestic data (age, sex, T classification, N classification, neck dissection, adjuvant chemo/radiotherapy, comorbidities, alcohol consumption, and type of TEP) in relation to the appearance of complications (Table 5). However, in the univariate analysis, none of the parameters taken into consideration were found to be statistically significant as complication risk factors.

## 4. Discussion

Voice rehabilitation with TEP is currently considered the most effective approach for patients who have undergone total laryngectomy. Numerous studies demonstrate that the objectively measured voice quality in patients rehabilitated with TEP is superior to the esophageal voice quality [7,10,11]. However, few studies focus on the long-term maintenance of TEP and the management of long-term complications. In our study, we observed the occurrence of complications in 36.8% of cases, with 39.2% in primary TEP cases and 33.4% in secondary cases. The percentage of complications we found is comparable to the current literature, especially in the context of secondary TEP, with reported rates ranging from 22% to 38.2% [12,13,14,15,16]. Notably, Scherl et al. [17] reported a higher complication incidence of 65.2% in 112 cases of primary TEP. The types of complications we observed are similar to those reported by other authors and include prosthesis leakage, granulation tissue formation, fistula leakage, and prosthesis extrusion. In our study, in 71.4% of cases, the complication was resolved through prosthesis replacement, granulation removal, prosthesis repositioning, or other interventions. Our study did not consider major post-surgical complications such as tracheal stenosis, wound infection, and pharyngocutaneous fistula arising from the primary or secondary implantation of the prosthesis. This decision was made because the goal of this study was to detect the appearance of complications over time after treatment stabilization. We found no differences in the occurrence of complications between primary and secondary TEP, consistent with Boscolo Rizzo et al.’s findings [18]. However, prosthesis extrusion and fistula leakage were only observed in primary TEP cases. Even Barauna Neto et al., in their systematic review similarly to our study, found a lower risk of fistula leakage in patients undergoing secondary TEP 2 compared to patients undergoing primary TEP [19]. The tendency towards extrusion or displacement of the prosthesis implanted via primary TEP may be attributed to its final position being dependent on the scar outcomes of the esophageal suture. Our study revealed a TEP failure rate of 35.7% among patients with complications and an overall failure rate of 13.1% across all patients. Our results showed that, patient abandonment (40%) and spontaneous closure (40%) were the leading cause of failure. Our observed failure rate aligns closely with findings from other authors [16,17,18,19,20,21], both in studies focusing on primary and secondary TEP. Additionally, in agreement with Boyd et al. [16], no clinical or anamnestic factor correlated with the onset of complications was identified. The high rate of voice prosthesis abandonment by patients who did not recognize themselves in the vocal tone obtained through the implantation of a phonatory prosthesis with both primary and secondary TEP confirms our previous study’s findings [7]. Despite objective assessments demonstrating a superior voice quality with TEP, the subjective evaluation by patients did not align with the objective findings. As indicated by the analyses of the voice handicap index and voice-related quality of life, the objectively observed good voice quality did not result in a statistically significant difference between patients rehabilitated with TEP and those with esophageal voice [7]. In our study, the reasons for abandonment are attributed to the patient’s inability to adapt to rehabilitation protocols, leading to persistent aphonia, or rejection of the new vocal tone. Additionally, 51 patients, as shown in Table 3, underwent frequent and closely spaced prosthesis replacements, with an average time of less than 3 months, to address complications. In light of the findings that emerged from our study, we believe that in the choice of vocal rehabilitation for patients who have undergone total laryngectomy, it is essential to provide comprehensive information to the patient about all available phonatory rehabilitation options. This information should be conveyed through group sessions, including for individuals rehabilitated with different methods (voice prosthesis or esophageal voice), ensuring more informed consent is obtained from the patient regarding their preferred method of rehabilitation. The rehabilitation of laryngectomized patients must be addressed using a multidisciplinary team that considers the personalities, personal needs, and the types of social relationships of individual patients in order to determine and apply the phonatory rehabilitation method most suitable for achieving a better quality of life. However, the study has some limitations, primarily due to the lack of consideration of the role of esophageal reflux and peri-fistular mucosal biofilm in the onset of complications. Nevertheless, the primary objective of the study was to assess the incidence of long-term complications and their management.

There are not many studies in the literature that take into account the complications of primary and secondary voice prosthesis simultaneously, as ours does. Our study has highlighted the necessity of informing the patient, through a multidisciplinary team, about the various modalities of phonatory rehabilitation, in light of cases of abandonment and spontaneous closure. We also highlighted how the types of complications, recorded over time, differ between primary and secondary TEPs.

The study’s strengths lie in the homogeneity of the clinical characteristics of the selected patients, who had not undergone prior treatment and had undergone primary laryngectomy.

## 5. Conclusions

Our results suggest that both primary and secondary TEP are valuable solutions for voice rehabilitation after TL. It may be worthwhile making an effort to identify prognostic factors that could severely influence TEP success and initiate, in these cases, a vocal rehabilitation approach with ES as a first step, reserving TEP as a secondary option.

## Figures and Tables

**Table 1:** Patients clinical-demographic data.

Data	Patients n. 76 (%)
**Age**	
Mean ± SD (years)	64.52 ± 11.3SD
**Follow-up**	
Mean ± SD (months)	42.42 ± 18SD
**Sex**	
M	66 (86.8)
F	10 (13.1)
**T classification**	
T3	40 (52.6)
T4	36 (47.3)
**N classification**	
N0	39 (51.3)
N+	39 (51.3)
**Neck dissection**	
No	7 (9.2)
Yes	69 (90.7)
**Adjuvant chemotherapy**	
No	61 (80.2)
Yes	15 (19.7)
**Adjuvant radiotherapy**	
No	44 (57.8)
Yes	32 (42.1)
**Comorbidities**	
No	19 (25)
Yes	57 (75)
**Alcohol habit**	
No	55 (72)
Yes	21 (27.6)
**TEP**	
Primary	46 (60.5)
Secondary	30 (39.4)

SD: standard deviation; T classification: tumor classification; N classification: node classification; TEP: tracheoesophageal puncture.

**Table 2:** Clinical and anamnestic data of patients with primary and secondary TEP.

Data	Primary TEP (46 Patients) (%)	Secondary TEP (30 Patients) (%)	*p* Value
**Age**			
Mean (year) ± SD	66.8 ± 11.5	61.0 ± 10.2	0.02
**Follow-up**			
Mean (month) ± SD	38.5 ± 18.4	48.36 ± 15.7	0.01
**Sex**			
Male	37 (80.5)	29 (97)	
Female	9 (19.5)	1 (3)	0.07
**T classification**			
T3	28 (60.8)	12 (40)	
T4	18 (39.2)	18 (60)	0.10
**N classification**			
N0	23 (50)	16 (53.4)	
N+	23 (50)	14 (46.6)	0.81
**Neck dissection**			
No	4 (8.7)	3 (10)	
Yes	42 (91.3)	27 (90)	1.00
**Adjuvant chemotherapy**			
No	39 (84.8)	22 (73.4)	
Yes	7 (15.2)	8 (26.6)	0.22
**Adjuvant radiotherapy**			
No	27 (58.7)	17 (56.6)	
Yes	19 (41.3)	13 (43.4)	1.00
**Comorbidity**			
No	9 (19.6)	10 (33.3)	
Yes	36 (78.4)	20 (66.7)	0.18
**Alcohol habit**			
No	32 (69.6)	23 (76.6)	
Yes	14 (30.4)	7 (23.4)	0.60

**Table 3:** Time complications occurrence and substitution TEP.

N. Patients	Type of TEP	Meantime Complication Months ± SD	Time Substitution Interval			
			≤3 Months		>3 Months	
			N	(%)	N	(%)
46	primary	3.51 ± 5.5	32	(69.6)	14	(39.4)
30	secondary	3.76 ± 7.7	19	(63.3)	11	(36.7)
*p*		0.65	0.57			

TEP: tracheoesophageal puncture; p: statistical significance < 0.05.

**Table 4:** Type of complications that occurred in the 28 patients.

Causes	N. Patients	%	Primary TEP (%) N (%)	Secondary TEP (%) N (%)	*p*
Prosthesis leakage	9	32.2	5 (27.8)	4 (40)	0.68
Fistula leakage	2	7.2	2 (11.1)	0 (0)	0.08
Granulations	5	17.8	3 (16.7)	2 (20)	0.54
Prosthesis extrusion	4	14.2	4 (22.2)	0 (0)	0.008
Spontaneous closure	4	14.2	2 (11.1)	2 (20)	0.52
Abandonment	4	14.2	2 (11.1)	2 (20)	0.52

TEP: tracheoesophageal puncture.

**Table 5:** Correlation between clinical–anamnestic data and complications.

Variables	No Complications N. 48 Patients		Complications N. 28 Patients		*p* Value
	N	(%)	N	(%)	
**Age**					0.63
≤65 yrs	25	(52.1)	13	(46.4)	
>65 yrs	23	(47.9)	15	(53.6)	
**Sex**					0.35
M	43	(89.6)	23	(82.1)	
F	5	(10.4)	5	(17.9)	
**cT**					0.19
T3	28	(58.3)	12	(42.9)	
T4	20	(41.7)	16	(57.1)	
**cN**					0.86
N0	25	(52.1)	14	(50)	
N+	23	(47.9)	14	(50)	
**Neck dissection**					0.24
No	3	(6.3)	4	(14.3)	
Yes	45	(93.8)	24	(85.7)	
**Adjuvant RT**					0.91
N0	28	(58.3)	16	(57.1)	
Yes	20	(41.7)	12	(42.9)	
**Adjuvant CT**					0.14
No	41	(85.4)	20	(71.4)	
Yes	7	(14.6)	8	(28.6)	
**Comorbidities**					0.58
No	11	(22.9)	20	(71.4)	
Yes	37	(77.1)	8	(28.6)	
**Alcohol habit**					0.35
No	33	(68.8)	22	(78.6)	
Yes	15	(31.3)	6	(21.4)	
**TEP**					0.61
Primary	28	(58.3)	18	(64.3)	
Secondary	20	(41.7)	10	(35.7)	

Yrs: years; M. male; F. female; cT: clinical tumor; cN: clinical node; RT: radiotherapy; CT: chemotherapy; TEP: tracheoesophageal puncture. *p* = 0.05.

## Data Availability

Data are contained within the article.
